# The Influence of Highly Aspherical Lenslets on Choroidal Thickness and Axial Length

**DOI:** 10.3390/jcm14197059

**Published:** 2025-10-06

**Authors:** Larissa Paulasto, Céline Carré, Martin Loertscher

**Affiliations:** 1 Institut für Optometrie, Fachhochschule Nordwestschweiz, Riggenbachstrasse 16, 4600 Olten, Switzerland; larissa.paulasto@gmail.com (L.P.); celine.carre@shinternet.ch (C.C.); 2 ELZA Institute Bahnhofplatz 15, 8001 Zurich, Switzerland; 3 Visilab SA, Hauptgasse 34, 4600 Olten, Switzerland; 4 Augeninstitut Aarau, Optometriepraxis Dr. Loertscher GmbH, Bahnhofstrasse 24, 5000 Aarau, Switzerland

**Keywords:** Myopia progression, Choroid, highly aspheric lenslets, Myopia control, axial length, full-field defocus, myopic defocus, peripheral refraction

## Abstract

**Objectives:** Recent studies have shown that highly aspheric lenslets (HAL) are effective in slowing myopia progression. Moreover, research indicates that an enhancement in choroidal thickness might serve as a biomarker for evaluating the efficacy of myopia control treatments. Therefore, this study examined the short-term effects of HAL and full-field +3.00 diopters (D) myopic defocus on sub-foveal choroidal thickness (SFCHR). **Design:** Prospective experimental study. **Participants:** Twenty-five participants aged 20–30 (mean 24.56 ± 2.467) years with a refraction error of emmetropia to −5.0 D (mean −2.255 ± 1.514 D). The contralateral non-dominant eye was used as control for each participant. **Methods:** The participants watched a movie projected at 6 m for 90 min on two separate occasions while wearing HAL or +3 D full-field myopic defocus lenses on their dominant eye. The control eye wore only a single-vision contact lens with the best-corrected distance vision. Three measurements of AL and SFCHR were captured before defocus, and after 60 and 90 min of defocus for both eyes. The main outcome measures were changes in SFCHR and AL over time. **Results:** Exposure to HAL and +3 D myopic defocus significantly increased SFCHR in the defocused eyes compared to the baseline (*p* < 0.001). The increase in SFCHR was 6.62 ± 6.32 µm with the HAL intervention and 7.36 ± 8.83 µm with the +3 D intervention. The difference between the two interventions was not statistically significant (*p* = 0.595). A significant mean difference of 3.176 ± 1.318 µm in SFCHR increase was observed with +3.00 D full-field defocus in the defocused eyes compared to the control eyes over the measurement period (*p* = 0.020). **Conclusions:** Short-term exposure to full-field myopic defocus increased choroidal thickness, which was comparable with that observed in peripheral myopic defocus with HAL, although the effect appears less pronounced.

## 1. Introduction

Myopia, also known as nearsightedness, is a refractive error that commonly results from excessive axial growth of the eyeball. An overly curved cornea and/or crystalline lens with increased optical power can also cause myopia. In the last few decades, myopia control has become a significant part of ophthalmic research and practice, which aims to halt the progression of myopia in children and prevent myopia-related eye diseases and blindness. Various myopia control treatments are available, such as orthokeratology, progressive lenses, soft-multifocal contact lenses, and the administration of atropine [1,2]. The latest myopia control treatment is the use of special spectacle lenses. Two types of lenses have successfully been introduced: highly aspherical lenslets (HAL) [3] and defocus-incorporated multiple segments (DIMS) [4] lenses. Both lenses are based on the potential principle of peripheral myopic defocus [5]; they induce a myopic defocus in the peripheral retina, in contrast to traditional correction methods that induce hyperopic peripheral defocus. This myopic defocus is hypothesized to provide a “stop” signal for excessive axial length (AL) growth.

The HAL lenses consist of a central zone with the usual refractive correction and 11 rings of continuous lenslets with a diameter of 1.1 mm [6]. According to the manufacturer these small lenslets produce “a volume of myopic defocus image with 0.7 mm depth and approximately 1.2 mm before the retina,” thereby following the peripheral retinal defocus theory. A two-year randomized clinical trial by Bao et al. [7] examined the progression of myopia in three intervention groups wearing either HAL, SAL (slightly aspherical lenslets), or single-vision lenses. The results showed that the HAL lenses significantly slowed spherical equivalent by 0.99 D and axial elongation by 0.41 mm compared to single-vision lenses. The SAL also showed a significant reduction in myopia progression compared to single-vision lenses; however, they had a lower efficacy in slowing myopia progression compared to HAL. This may demonstrate the dose-dependent relationship seen in other myopia interventions, where higher amounts of peripheral defocus show greater results in stopping myopia [7]. Notably, these results indicate that a minor change in asphericity can result in such a significant effect on AL elongation. Another recent long-term study on HAL lenses evaluated its effect on choroidal thickness [8]. Overall, the results demonstrated that children wearing HAL lenses experienced an inhibitory effect on choroidal thinning over three years compared to children wearing traditional single-vision lenses. In addition, they observed less AL progression in the HAL group than in the single-vision group. Thus, this study partially (not in all groups) established a correlation between AL and choroidal thickness changes, leading to the assumption that choroidal changes are not the only factor involved in myopia control.

While studies on HAL lenses have demonstrated effective results in preventing the long-term progression of myopia [3] and some choroidal thickness changes [8], their effect over short periods remains unknown. Several short-term studies have shown that a monocular myopic defocus induced with single-vision lenses led to choroid thickening and AL shortening over a period of 30 min to 2 h [9]. In contrast, hyperopic defocus using minus-powered single-vision lenses results in reciprocal changes, leading to choroidal thinning and axial elongation, wherein AL and choroidal thickness are negatively correlated. This is because the change in choroid thickness moves “the retina towards the plane of focus” [10]. Consequently, a positive choroid thickening decreases the axial length. The exact mechanism of the compensatory response by the choroid during defocus is not fully understood. However, Swiatczak, Schaeffel, and Calzetti [9] showed increased choroidal blood flow after 30 min of monocular myopic defocus of +2.5 dpt. They hypothesized that myopic defocus activates a retinal signaling cascade that ultimately increases choroidal blood flow and inhibits ocular growth. These signaling cascades may correlate with the feedback loop present in the emmetropization process [11].

The duration of refractive defocus ranges from 30 min to 12 h in all studies, which report significant changes in choroidal thickness in the affected eye [12,13,14,15,16,17,18,19]. Studies using a duration of 30 min to 2 h had patients watch a movie or documentary under dim light conditions (approximately 10 lux) [18,20]. Chakraborty, Read and Collins [18] used 12 h and had patients perform their usual tasks throughout the day and reported that choroidal thickness and axial length vary throughout the day. Similarly, Moderiano et al. [20] also presented patients with monocular myopic defocus (+3.0 dpt) for 2 h once in the morning and once in the evening and found a greater reduction in axial length and a greater increase in subfoveal choroidal thickness in patients after 2 h of movie viewing in the evening compared to that in the morning.

Numerous studies have demonstrated that monocular myopic defocus induced by single-vision lenses results in an increase in sub-foveal choroidal thickness (SFCHR) and a reduction in axial length (AL). This study seeks to replicate these findings using single-vision lenses with a power of +3.00 diopters (D) and to additionally assess the effects of HAL lenses, a comparison that has not been previously undertaken.

## 2. Materials and Methods

### 2.1. Study Design and Participants

This prospective experimental study was conducted at the Institute of Optometry, FHNW, Olten, between 21 November 2023 and 27 March 2024. The study protocol was approved by the local ethics committee (EKNZ Studi Id: 2023-01882, 8 November 2023), and each participant has given their written consent to participate. The study was performed in accordance with the Declaration of Helsinki.

Twenty-eight young adults were recruited based on a power analysis derived from a pilot study (*n* = 9), which indicated a required sample size of 20 eyes (effect size = 0.3916, partial η^2^ = 0.133, α = 0.05, and power = 0.95). An additional eight participants were included to account for potential dropouts or data exclusion. Two of the 28 participants were excluded due to insufficient choroidal visibility on the optical coherence tomography (OCT), which made it impossible to measure the choroid. Another participant was excluded for only attending one appointment instead of the required two. Therefore, 25 participants (men: 10, women: 15) were finally analyzed. The participants wore their usual fitted contact lenses during the study. The inclusion criteria for this study were healthy participants aged 20 to 30 years (mean 24.56 ± 2.467) with a refractive error ranging from emmetropia to −5.00 D (mean −2.255 ± 1.514 D). Participants were required to have either no astigmatic correction or astigmatic correction of ≤−1.50 D in their contact lenses (mean: −0.14 ± 0.335 D), while presenting a visual acuity of LogMar 0.00 or better. This is because high astigmatism could potentially result in poor imaging of the choroid and incorrect measurements. Each participant presented a visual acuity of LogMar 0.00 or better (mean −0.05 ± 0.9) in their contact lenses. The sensory-dominant eye was determined in each participant and received the monocular defocus. Overall, 72% and 28% of the participants presented dominance in the right and left eye, respectively. None of the participants had binocular problems, such as strabismus, anisometropia, amblyopia, or retinal or choroidal disorders.

### 2.2. Measurement Procedure

**Figure 1:**
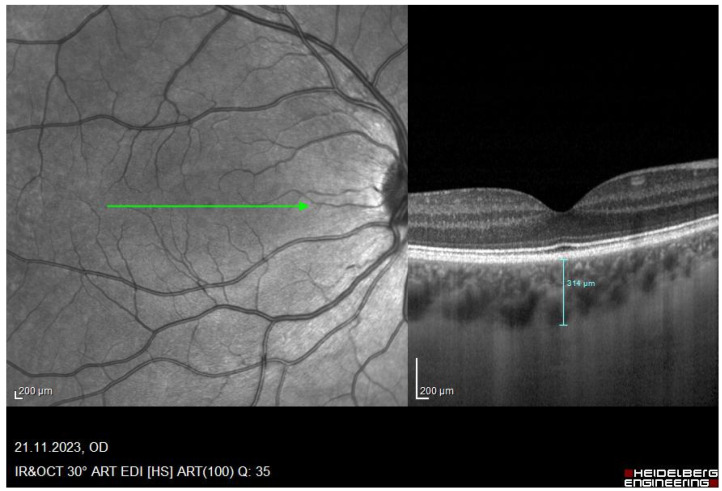
Red-free image of the retina and its corresponding OCT image of a participant with a manual segmentation line drawn through the SFCHR. A SFCHR of 314 um was measured in this particular subject. Image captured with the Spectralis OCT.

Each participant was required to attend two appointments, once wearing a myopic defocus of +3.00 D and once wearing HAL lenses. The minimum washout period between these visits was five days. At the first appointment, the type of defocus, HAL or +3.00 D, that the participant would receive first was randomly determined. At the second appointment, the participant was given the other defocus. Both appointments for all participants took place between 12:00 pm to 4:00 p.m. At the first appointment, visual acuity and the sensory dominant eye were determined. The randomly assigned lens (either HAL or +3.00 D) was then monocularly fitted to the patient’s dominant eye. The SV +3.00 D or HAL lenses were fixed to plastic frames. Each lens was individually centered on the participants’ monocular pupil distance. A variety of plastic frames were available to ensure comfort and correct lens centration. During the defocus period, participants viewed a TV-show for 90 min using a projector, which projected at 6 m. The luminance of the room was 10 lux (mean 10.277 ± 0.669). A maximum of six participants were examined at each appointment to ensure the same viewing distance of 6 m for all participants. Three measurements of AL and sub-foveal choroidal thickness (SFCHR) were obtained from the defocused and non-defocused eyes to determine the effect of myopic defocus. The first measurements were taken before defocus was applied. The second and third measurement were taken after 60 min and 90 min of viewing, respectively. The non-defocused eye was used as a control. Spectacles with the defocus were only removed right before each measurement to avoid clear vision. Three consecutive measurements were taken at each measurement time point. AL was measured using the Lenstar LS 900 (Haag Streit AG, Köniz, Switzerland) optical biometer from which automatically captures three consecutive measurements. SFCHR was measured using the SPECTRALIS OCT (Heidelberg Engineering GmbH, Heidelberg, Germany) with a one-line scan 15° (compromised with 100 averaged scans) horizontally through the macula in the enhanced depth imaging (EDI) mode. The SFCHR was first manually segmented and measured using Heidelberg software [Figure 1].

The analysis was performed individually by the two investigators (L.P. and C.C.) to ensure the verifiability of the values. The average values were considered in case of any deviation.

### 2.3. Statistical Analysis

All statistical analyses were conducted using Microsoft Excel, GPower (Version 3.1) and IBM SPSS (Version 28). Graphs were computed with IBM SPSS. The interobserver agreement of the SFCHR was analyzed using a Bland–Altman Plot. Reliability analysis was performed using a two-way mixed-effects model and absolute agreement definition to obtain an intraclass correlation coefficient (ICC) for the measurement by the two investigators and for the three repeated SFCHR measurements. Descriptive statistics were presented as mean ± standard deviation. A Shapiro–Wilk test was used to assess normality. A paired t-test was used to compare the baseline measurement between the right and left eye for SFCHR and AL. Repeated measures analysis of variance (ANOVA) with within-subjects (three-time points: baseline, 60 min, and 90 min) and between-subject designs (Intervention: HAL or +3.00 D) were used to evaluate the effects of the intervention over time and assess potential differences between the two intervention groups. Pearson’s correlation coefficient was to determine a correlation between the AL, SFCHR baseline measurements and refractive error and between refractive error and change in SFCHR and AL. Statistical significance was set at *p* < 0.05.

## 3. Results

This study included 25 participants (men: 10, women: 15) aged between 20 and 30 (mean 24.56 ± 2.467) years with a refraction error of emmetropia to −5.0 D (mean −2.255 ± 1.514 D).

### 3.1. Measurement Reliability and Concordance

**Figure 2:**
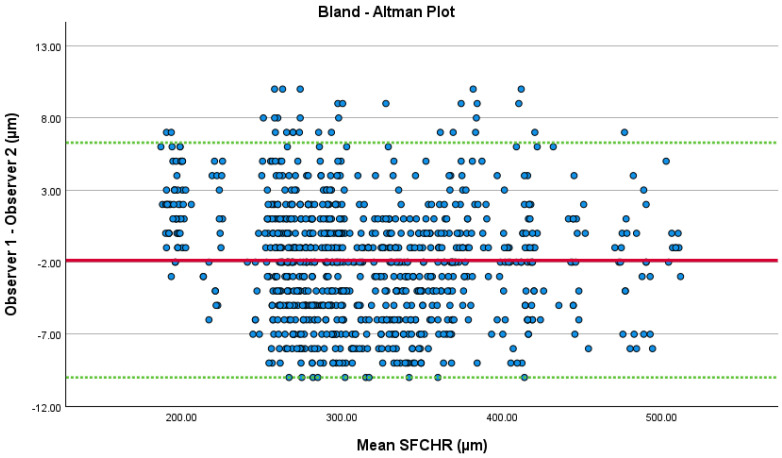
Bland–Altman Plot: The green lines represent 1.96 × standard deviations and the red line represents the mean difference between the two SFCHR measurements.

The Bland–Altman plot showed good agreement for choroidal thickness change between the two investigators, with most differences falling within the confidence intervals [Figure 2]. The mean difference was −1.80 ± 4.243 µm. The regression analysis revealed a low R^2^ value of 0.05 and no significant relationship between the differences and the mean.

An ICC of 0.999 was observed in the reliability analysis between the measurements of the two investigators, indicating that both investigators were in agreement. The ICC between the three consequent SFCHR measurements (averaged from the values from the two investigators) also had a value of 0.999. While these results suggest very high reliability, such values are uncommon in biological measurements. This may be explained by the relative homogeneity of the sample, as choroidal thickness values were similar across many patients, and by the large number of measurements (n = 450), which stabilized the ICC estimate. In addition, the choroid itself is relatively thick (mean: 313.96 ± 67.07 µm), making differences of less than 10 µm negligible compared with the mean, thereby inflating the ICC. Nevertheless, these reliability metrics should be interpreted with caution, as the Bland–Altman plot provides a more informative assessment of agreement.

### 3.2. Baseline Data

The Shapiro–Wilk test showed a normal distribution for all measurements of AL, SFCHR and spherical equivalent refractive error (SER). Paired t-tests showed that, at the baseline measurement, no significant difference was present between the right and left eye regarding SFCHR and AL. At baseline, a mean SFCHR of 313.96 ± 67.07 µm was observed for both eyes in all participants. The mean AL at baseline for both eyes was 24.54 ± 0.944 mm. Additionally, a paired t-test was performed to assess the reliability of the baseline measurement during the different appointments. No significant difference was observed in AL (*p* = 0.642) and SFCHR (*p* = 0.279) measurements at baseline. The SER between the right and left eye was not significantly different (*p* = 0.428), and the mean SER for both eyes was −2.325 ± 1.511 dpt. Pearson’s correlation revealed that the refractive error was significantly correlated with SFCHR (*p* = 0.017) and AL (*p* < 0.001), demonstrating that individuals with higher myopia have thinner choroids and longer ALs. Despite the expectation of a correlation between AL and SFCHR, this study did not observe one (*p* = 0.060). This may be due to the small sample size.

### 3.3. Effect of HAL and +3.00 D Lenses

The change in SFCHR and AL between the baseline and the two time points was calculated to assess the effect of HAL and +3.00 D lenses. The mean change in SFCHR and AL compared to baseline alone was calculated as shown in Table 1.

**Figure 3:**
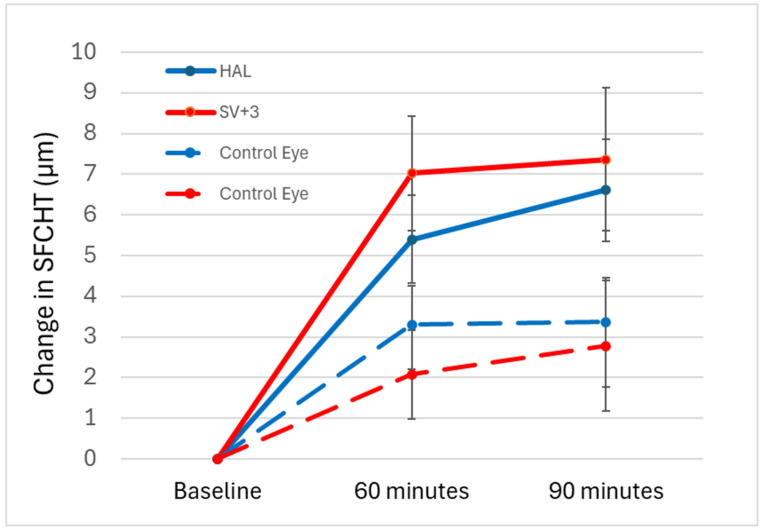
Change in SFCHR over time in the four groups: +3.00 D-defocused (solid red) and control (dashed red). HAL-defocused (solid blue) with control eyes (dashed blue). Error bars indicate ± standard error (SE).

A within-subjects repeated measures ANOVA revealed that exposure to HAL and +3.00 D myopic defocus caused significant differences in SFCHR among the different time points in the defocused eyes (*p* < 0.001). Figure 3 illustrates the overall change in SFCHR over time. In the defocused eye, +3.00 D caused slightly more thickening (7.36 ± 8.83 µm) than HAL (6.62 ± 6.32 µm) after 90 min, although this was not statistically significant (*p* = 0.595). In contrast, pairwise comparisons with Bonferroni adjustment showed significant differences between baseline measurements and after 60 and 90 min in both interventions (*p* < 0.001). However, no significant difference was observed in either intervention between 60 and 90 min (*p* = 0.482 for HAL and *p* = 1.0 for +3.00 D).

In the control eye, there was a slightly greater thickening when the fellow eye was exposed to HAL (3.38 ± 5.33 µm) compared to +3.00 D (2.78 ± 8.01 µm) after 90 min. However, similarly to the defocused eye, this difference was not statistically significant (*p* = 0.734). Pairwise comparisons with Bonferroni adjustment revealed no significant difference between baseline and 60 min or 90 min in the +3.00 D intervention (baseline vs. 60 min: *p* = 0.151, baseline vs. 90 min: *p* = 0.140). Conversely, a significant difference of 3.300 ± 4.845 was noted between baseline and 60 min in the HAL intervention (*p* = 0.08). However, the difference between baseline and 90 min was not statistically significant (*p* = 0.050). Additionally, no significant difference was observed between 60 and 90 min (*p* = 1.00 for both interventions). A significant mean difference of 3.176 µm (SE = 1.318 µm) was observed in the SFCHR with +3.00 D when comparing defocused eyes with control eyes over the entire measurement period (*p* = 0.020). However, the mean difference of 1.779 µm (SE = 0.960 µm) for HAL between defocused and control eyes did not reach statistical significance (*p* = 0.070).

**Figure 4:**
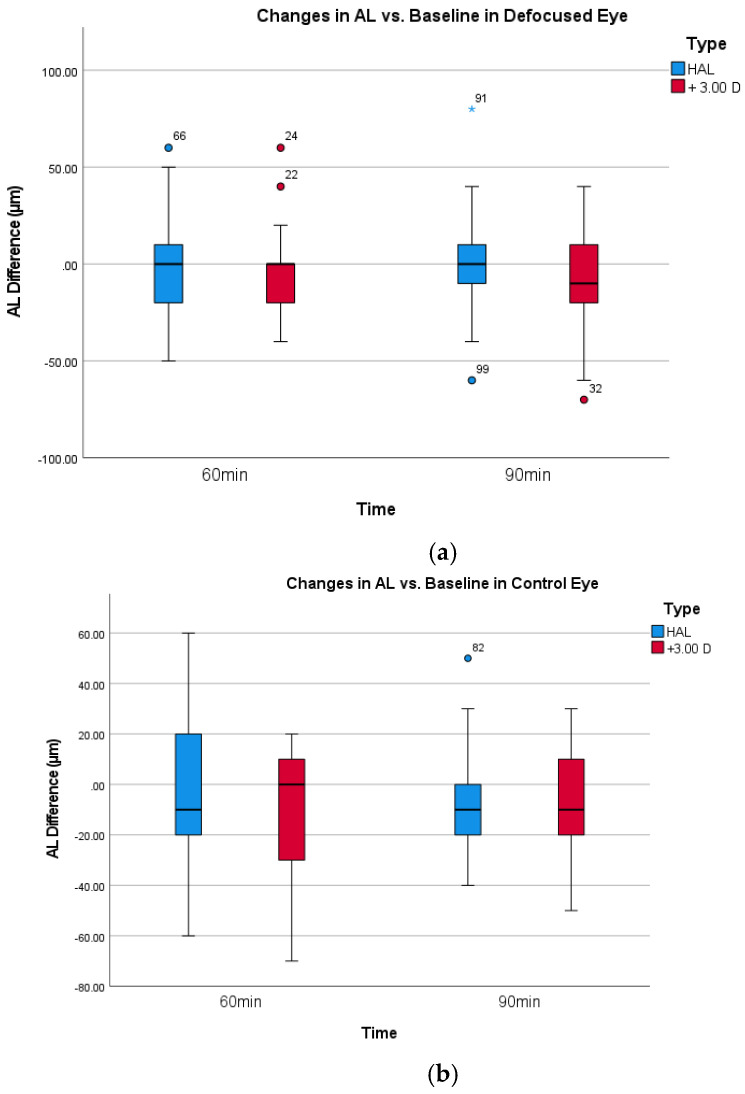
(**a**)**.** Box-plot change in AL for defocused eyes. (**b**)**.** Box-plot change in AL for the non-defocused control eyes.

In contrast to the significant results for SFCHR, neither the change in axial length for HAL (0.00 ± 28.28 µm) nor full defocus with +3.00 D (−7.600 ± 26.81 µm) reached a significant level. Repeated ANOVA measures revealed no significant within-subject effect of time for AL across both intervention groups in the defocused and control eyes (defocus group: *p* = 0.554, control group: *p* = 0.148). Figure 4a,b shows the change in AL over time. As expected, all pairwise comparisons with Bonferroni adjustment showed no significant difference in AL between all the time points in both eyes and interventions.

Additionally, Pearson’s correlation analysis did not observe a significant correlation between refractive error and SFCHR change over time, either in the eyes defocused with the interventions (HAL: PCC = −0.235, *p* =0.26/+3.00 D: PCC = 0.382, *p* = 0.59) or in the control eyes (HAL: PCC = −0.265, *p* = 0.20/+3.00 D: PCC = 0.153, *p* = 0.47). No correlation analysis was performed for AL, as no significant change was observed during the overall analysis in repeated ANOVA measures.

## 4. Discussion

Our findings demonstrate that full-field myopic defocus of +3.00 D and peripheral myopic defocus with HAL lenses cause choroidal thickening in the affected eye after 60 min. However, our results did not demonstrate AL shortening in both interventions, and found no correlation between changes in SFCHR and AL.

Exposing the retina to optical myopic defocus induces an increase in choroid thickness [13,15,16], which disrupts the diurnal choroidal thickness variation [18]. The choroid thickness also changes in response to astigmatic defocus, depending on the presented axis [21]. However, a previous study reported no changes in choroidal thickness due to optical defocus [12]. In contrast, our results show an increase in SFCHR of +7.36 ± 8.83 µm with +3.00 D myopic defocus after 60 min but no further change in SFCHR thereafter. This is comparable with the +12 ± 3 µm increase using a +3.00 D myopic defocus reported by Sander, Collins and Read [13], and the +10 ± 5 µm increase using +2.00 D myopic defocus reported by Chiang, Chen and Phillips [14].

More complex optical lenses for myopia control, such as the DIMS lens [4] and the HAL lens, [22] with its multiple optical segments on the optical surface, are thought to slow the progression of myopia by applying relative myopic defocus to the peripheral retina. A recent study showed that the SFCHR increased after 1 week +6.75 ± 1.52 µm and reached a maximum after 12 months of +13.64 ± 2.62 µm by having the subjects wear the DIMS lenses [23]. In another study with HAL [8], the choroidal thickness was analyzed over a two-year period and showed an increase, although it was not linear. Similarly to choroidal thickness, the SFCHR increased from baseline to 12 months, reduced again and increased thereafter. Meanwhile, the control group wearing single vision glasses showed a significant reduction in SFCHR [8]. Similarly, our results showed an increase of +6.62 ± 6.32 µm in SFCHR after 60 min, which is consistent with the results of previous studies [8,23]. Therefore, the short-term changes in SFCHR with multisegmented optics may be in correspondence with the long-term effect. However, a recent study showed that when the multisegmented optic is used with +3 D positive and −3 D negative lenslets, myopia progression is slowed down over a one-year period [24]. Unfortunately, change in SFCHR was not measured in this study, therefore a direct comparison of our SFCHR results is not possible. Nevertheless, our hypotheses that myopic defocus slows the progression of myopia by inducing choroidal thickening whereas hyperopic defocus promotes myopia progression and most likely reduces the thickness of the choroid contradicts their results.

Given that myopia progression can also be slowed down with negative powered lenslets, it is important to consider whether the term ‘diopters’ is important for optical myopia control systems [24]. Specifically, studies should evaluate whether the effectiveness of optical methods in the peripheral retina actually depends on a diopter value, since a recent optical analysis of the DIMS and the Stellest lens revealed that the change in relative peripheral refraction is low [25,26]. This highlights the need to determine whether this phenomenon is indicative of a change in high-order aberration [27,28] or, alternatively, a change in contrast perception [29]. Moreover, this phenomenon may be attributable to the differential ratio between central clear foveal visual acuity and peripheral blur. Additionally, it could also explain why the efficacy of orthokeratology in slowing myopia progression is somewhat dependent on pupil size [30,31] and more complex corneal molding pattern [32].

No significant change was observed in the AL. Consequently, a significant correlation between the change in SFCHR and AL could not be observed either. These results are consistent with those of studies that were able to observe a significant change in AL, which also noted a negative correlation between SFCHR and AL [9,13,19]. Previous studies evaluated the effects of myopic and hyperopic defocus on AL and SFCHR and found contrasting effects with hyperopic defocus compared to myopic defocus; elongation of AL and SFCHR thinning was observed with hyperopic defocus. These changes range from −3 ± 14 µm to −10 ± 5 µm for SFCHR and +8 ± 14 µm to +9 ± 9 µm for AL [13,14,16,33].

Our results indicate that no further increase after 60 min was observed in either intervention, which indicates that choroid thickening will eventually reach a plateau. This is consistent with the results of a previous study, where the choroid increase in thickness plateaued at 40 min for emmetropic eyes and 25 min for myopes [16]. Once the plateau was reached, the SFCHR stayed relatively the same in both groups. Sander, Collins, and Read [13] also speculated that the choroid has a limited capacity to thicken in the short term, since they did not observe an additive effect of homatropine on myopic defocused SFCHR, even though homatropine and myopic defocus cause a similar SFCHR increase.

Additionally, no evident significant change in SFCHR was observed when comparing the results of different time points in the control eye. These observations align with previous studies [14,18,19], which also only found a significant change in SFCHR in the affected eye and not in the other eye.

Moreover, no correlation was observed between refractive error and change in SFCHR or AL. This finding is consistent with the findings of several studies which did not observe such a correlation [13,16,19]. However, Swiatczak, and Schaeffel [34] found that myopes tended to develop longer AL after being exposed to positive defocus, whereas AL shortening is observed in emmetropes, suggesting that myopic eyes have a “reduced ability to detect positive defocus”. This singular study does not align with the studies mentioned above, which suggests that emmetropic and myopic eyes respond similarly to defocus. Nevertheless, this study was able to demonstrate the overall relationship between refractive error and SFCHR and AL, which is consistent with the literature reporting that myopes have longer ALs and thinner choroids [35,36].

Despite the positive outcomes, the current study has some limitations. First, the 90 min TV-show used various sounds, lighting, and visual elements, which could have potentially caused different emotions in the participants, which may have affected blood pressure and blood flow, and ultimately affect choroidal thickness. In contrast, previous studies ensured that the participants did not perform certain activities before the appointments, ones that could possibly affect the choroidal thickness. These included coffee consumption, vigorous exercise, and smoking [9,12]. Second, the non-defocused eye had clear vision throughout the study and acted as a control variable. Another control variable with no binocular defocus would have provided better insight and comparison for both groups of myopic defocus. This could also have raised awareness of the effect of the experimental condition (viewing distance, visual effects from the movie, time of the day) on AL and SFCHR. Third, recording the SFCHR after a recovery period would have provided additional information. For instance, Wang, Chun, Liu, Lee, Sun, Zhang, Lam, Liu, and To [15] measured the SFCHR and AL after a 2 h recovery period by applying a defocus for 2 h. They discovered that the SFCHR significantly thinned after the defocus was applied in the recovery period. What follows is that the effects of defocus are reversible. Fourth, all measurements were taken between 12:00 p.m. and 4:00 p.m. However, this time window may be too large to effectively minimize the possible effects of diurnal fluctuations [18,19]. Furthermore, cycloplegic refraction was not conducted before the measurements to ensure that all participants wore accurate refractive correction in their contact lenses and were not overcorrected with minus lenses. This study only checked the refractive correction through the desired, sufficient visual acuity. Finally, the study cohort only included young adults, to obtain better compliance from the participants. However, the effects on children would be interesting, since they are the ones who are most likely prescribed to wear HAL lenses in practice. Therefore, further research is needed to investigate the effect of optical defocus and its interaction with the choroid.

## 5. Conclusions

In conclusion, this study demonstrated significant sub-foveal choroidal thickening when myopic defocus was applied. In addition, this study demonstrated that the use of peripheral myopic defocus through HAL causes choroidal thickening similar to that with a full-field myopic defocus of +3.00 D. Overall, no significant shortening of AL was observed, thus, no correlation was observed between the change in choroidal thickness and AL. These findings contribute to our understanding of the role of the choroid in controlling myopia and indicate that HAL have promising effects. Therefore, further research is required to validate these findings and explore new factors and treatments for halting myopia development.

## Figures and Tables

**Table 1:** Comparison of SFCHR and Al change from baseline to 60 min and 90 min defocus for full-field +3.00 D and peripheral defocus with HAL. Mean changes ± SD. Significant changes with *p* < 0.05 are marked with *, and significant changes with *p* < 0.001 are marked with **.

	Full-Field +3.00 D		HAL	
Defocused eye	SFCHR in µm	AL in µm	SFCH in µm	AL in µm
**Defocus 60 min.**	7.020 ± 7.041 **	−4.800 ± 22.93	5.400 ± 5.446 **	0.00 ± 25.00
**Defocus 90 min.**	7.367 ± 8.831 **	−7.600 ± 26.81	6.616 ± 6.321 **	0.00 ± 28.28
**Control eye**				
**Defocus 60 min.**	2.080 ± 5.495	−7.200 ± 24.41	3.300 ± 4.845 *	−2.00 ± 28.87
**Defocus 90 min.**	2.780 ± 8.006	−7.200 ± 21.51	3.380 ± 5.334	−5.200 ± 21.63

## Data Availability

The data that support the findings of this scientific publication are available from the corresponding author upon reasonable request.

## Abbreviations

The following abbreviations are used in this manuscript:

HAL	Highly Aspheric Lenslets
SFCHR	Sub-foveal Choroidal Thickness
AL	Axial Length
D	Diopter
OCT	Optical Coherence Tomography
